# miR-71 and miR-263 Jointly Regulate Target Genes *Chitin synthase* and *Chitinase* to Control Locust Molting

**DOI:** 10.1371/journal.pgen.1006257

**Published:** 2016-08-17

**Authors:** Meiling Yang, Yanli Wang, Feng Jiang, Tianqi Song, Huimin Wang, Qing Liu, Jie Zhang, Jianzhen Zhang, Le Kang

**Affiliations:** 1 Institute of Applied Biology, Shanxi University, Taiyuan, Shanxi, China and State Key Laboratory of Integrated Management of Pest Insects and Rodents, Institute of Zoology, Chinese Academy of Sciences, Beijing, China; 2 Beijing Institutes of Life Science, Chinese Academy of Sciences, Beijing, China; Howard Hughes Medical Institute, UNITED STATES

## Abstract

Chitin synthase and chitinase play crucial roles in chitin biosynthesis and degradation during insect molting. Silencing of Dicer-1 results in reduced levels of mature miRNAs and severely blocks molting in the migratory locust. However, the regulatory mechanism of miRNAs in the molting process of locusts has remained elusive. In this study, we found that in chitin metabolism, two crucial enzymes, chitin synthase (CHS) and chitinase (CHT) were regulated by miR-71 and miR-263 during nymph molting. The coding sequence of *CHS1* and the 3’-untranslated region of *CHT10* contain functional binding sites for miR-71 and miR-263, respectively. miR-71/miR-263 displayed cellular co-localization with their target genes in epidermal cells and directly interacted with *CHS1* and *CHT10* in the locust integument, respectively. Injections of miR-71 and miR-263 agomirs suppressed the expression of *CHS1* and *CHT10*, which consequently altered chitin production of new and old cuticles and resulted in a molting-defective phenotype in locusts. Unexpectedly, reduced expression of miR-71 and miR-263 increased *CHS1* and *CHT10* mRNA expression and led to molting defects similar to those induced by miRNA delivery. This study reveals a novel function and balancing modulation pattern of two miRNAs in chitin biosynthesis and degradation, and it provides insight into the underlying molecular mechanisms of the molting process in locusts.

## Author Summary

Molting is a crucial process in the growth and development in insects. Disturbing the molting process represents an attractive strategy for developing safe and effective insecticides. The migratory locust is a hemimetabolous pest that undergoes five molting stages in its life cycle. Similar molting defects can be observed in expression silencing of the key genes both in miRNA processing and in chitin metabolism. However, any link between a specific miRNA to chitin metabolism has not yet been established. In this study, we elucidated a mechanism by which two miRNAs regulate chitin metabolism related to the molting process. We found that miR-71 and miR-263 directly repress two genes, *chitin synthase1* (*CHS1*) and *chitinase10* (*CHT10*), which are required for chitin biosynthesis and degradation in chitin metabolism. Manipulation of miR-71 and miR-263 expression blocked molting and resulted in abnormal molting by negatively regulating the expression of *LmCHS1* and *LmCHT10*. Furthermore, both up-regulation and down-regulation of *LmCHS1* and *LmCHT10* by miRNA manipulation altered the chitin content of the new cuticle and old cuticles, leading to a similar aberrant molting phenotype. Our results demonstrate a balancing modulation pattern of two miRNAs in chitin biosynthesis and degradation that controlled the precise molting process in locusts.

## Introduction

Molting is a crucial process in insect growth and development [1,2]. Chitin, as a vital component of the cuticle of the epidermis, plays key roles in maintaining morphology and the molting process [3]. Because chitin is absent in plants and vertebrates, and insect growth and development are strictly dependent on chitin biosynthesis and degradation, chitin metabolism represents an attractive target for developing safe and effective insecticides [4].

The migratory locust *Locusta migratoria*, a worldwide insect pest species, undergoes five molting stages in its life cycle [5,6]. The chitin-mediated molting process is considered to depend on two crucial genes, *chitin synthase* (*CHS*) and *chitinase* (*CHT*), which are regulated by molting hormone 20-hydroxyecdysone (20E) and juvenile hormone [7,8,9,10,11]. Chitin synthases are the key regulatory enzymes for chitin synthesis in insects and represent a specific target of insecticides [12]. The *LmCHS1* gene cloned from the migratory locust is expressed specifically in the epidermis during the molting stage. Knockdown of the *LmCHS1* gene increases the number of non-molting and abnormal molting nymphs [6]. However, another paralog *LmCHS2* contributes to the biosynthesis of chitin associated with the peritrophic matrix [13]. Moreover, chitinases are hydrolytic enzymes that are required for the degradation of glycosidic bonds of chitin [14]. *TcCHT10* prevents larval molting and plays a vital role during the molting process at all developmental stages; the other paralogs, *CHT5* and *CHT7*, prevent molting and wings from folding properly only in adults [15,16]. An interesting feature of *CHS1* and *CHT10* in locusts is that the abrupt increase and decrease in transcript levels at the end of every nymph stage (before molting) suggest that the two key enzymes are likely precisely modulated in the molting process. However, the underlying regulatory molecular mechanisms of enzyme-dependent chitin metabolism and the molting process have remained elusive.

MicroRNAs (miRNAs), small non-coding regulatory RNAs, have emerged as key posttranscriptional regulators of gene expression in multiple biological processes [17] because they can directly trigger translational repression or mRNA degradation by low complementary base-pairing with the 3’UTRs of the target genes [18,19]. However, recent studies have shown that miRNAs can extensively target the protein-coding region of mRNAs in animals or insects [20,21,22]. Many studies have shown that miRNAs critically affect the molting of insects, thus resulting in molting defect phenotypes. For example, miR-8-5p and miR-2a-3p negatively regulate membrane-bound trehalase and phosphoacetylglucosamine mutase of the chitin biosynthesis pathway, leading to a significant reduction in survival rate along with a molting defect phenotype in the hemipteran insect *Nilaparvata lugens* [23]. Several distinct miRNAs have been approved in the regulation of insect metamorphosis. The loss of miR-2 up-regulates Kr-h1 mRNA, thereby leading to impaired metamorphosis [24,25]. Additionally, let-7 and miR-125 mutants induce temporal mis-regulation of specific metamorphic processes in *Drosophila* [26]. In the migratory locust, we reported that depletion of Dicer-1, the enzyme that catalyzes the final step of miRNA biosynthesis, induced a molting defect [27]. Results indicated that miRNAs play a crucial role in regulating the molting process of locusts. However, the mechanism regarding how miRNAs affect posttranscriptional modifications in the molting process has not yet been fully elucidated.

Considering that CHS1 and CHT10 are crucial molt-dependent enzymes that balance chitin metabolism in many insect species [15,16,28,29], we chose *CHS1* and *CHT10* as candidate genes. We hypothesized that miRNAs might play essential roles in the regulation of *CHS1*- and *CHT10*-mediated molting processes. In this study, we performed small RNA transcriptome sequencing to identify expressed miRNAs in the integument of locusts. We found that the integument-expressed miR-71 and miR-263 directly target the two key genes *CHS1* and *CHT10* and regulate chitin production during the molting process, resulting in the successful molting of the migratory locust. Our results reveal a molecular mechanism by which miRNAs play a role in balancing the modulation of *CHS1*- and *CHT10*-dependent chitin metabolism during molting.

## Results

### miRNA identification

**Fig 1:**
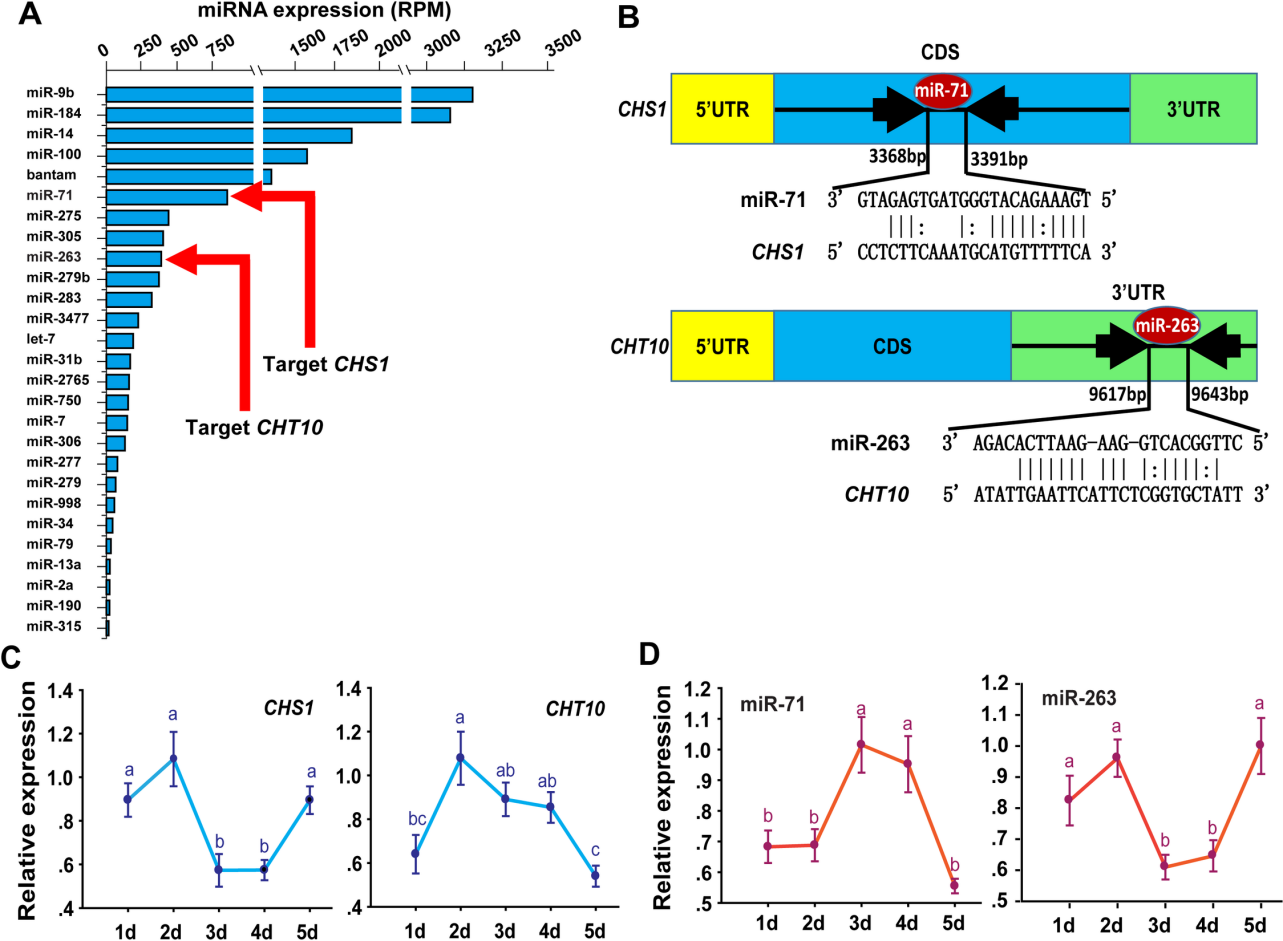
miR-71 and miR-263 may target *chitin synthase* (*CHS1*) and *chitinase* (*CHT10*), two key enzymes of chitin metabolism. (A) miRNAs identified in the locust integument by small RNA transriptome. Only the miRNAs with high/moderate expression level (reads per million, RPM>15) are shown. (B) miR-71 and miR-263 target sites were predicted in the *CHS1* and *CHT10* genes of *Locusta migratoria*. (C) The expression levels of *CHS1* and *CHT10* were determined in the integuments every day (D1–5: Days 1–5) during the second-instar nymph stage using qPCR. (D) The expression levels of miR-71 and miR-263 were determined in the integuments every day (D1–5: Days 1–5) during the second-instar nymph stage using qPCR. The qPCR data are presented as means ± SEM (*n* = 6); the same letter indicates data that are not significantly different.

To identify the miRNAs associated with molting, we sequenced a transcriptome of small RNAs of the locust integument, which is an important tissue during the molting process in insects. A total of 15,459,187 sequencing reads were obtained, of which 4,590,268 (29.7%) corresponding to mature and star strands were mapped to the known miRNA precursors of locusts [30]. Forty-five conserved miRNAs showed transcriptional activities (reads per million threshold 1) in the integument of locusts. Their expression levels varied over several orders of magnitude. The top ten most highly expressed miRNAs were miR-9b, miR-184, miR-14, miR-100, bantam, miR-71, miR-275, miR-305, miR-263 and miR-279b (Fig 1A). All of the expressed miRNAs were used for further miRNA candidate screening.

### Potential miRNAs targeting *CHS1* and *CHT10*

*CHS1* and *CHT10* involved in chitin metabolism have been confirmed to regulate the insect molting process [15,16,28,29]. Using the miRanda software, we predicted the expressed miRNAs that could potentially bind to *LmCHS1* and *LmCHT10*. Thirteen miRNAs exhibited potential target sites in the 3’UTR and CDS regions of *LmCHS1*, and 6 miRNAs possessed target sites located in the 3’UTR of *LmCHT10* in locusts (Fig 1B, S1 Table, and S2 Table). An additional prediction software, RNAhybrid, was used to further improve the target prediction efficiency. The RNAhybrid program also identified *LmCHS1* and *LmCHT10* as potential targets for miR-71 and miR-263, respectively (S1 Fig). Furthermore, we confirmed the absence of miR-71 and miR-263 binding sites in the *Tweedle*, *Cryptocephal*, *Obstructor*, *Knickkopf*, and *ecd1* genes to exclude the other possible miR-71/miR-263 targets [31,32,33,34,35], which can lead to molting defects similar to those caused by *LmCHS1* and *LmCHT10*.

To confirm the correlation of the expression pattern between *LmCHS1*, *LmCHT10* and their target miRNAs, we performed stem-loop quantitative reverse transcriptase-polymerase chain reaction (qRT-PCR) to quantify the expression levels of these predicted miRNAs and target genes in the integument of second-instar nymphs (S2 Fig). The overall expression of miRNAs (miR-71 or miR-263) and that of the target genes (*LmCHS1* or *LmCHT10*) exhibited opposite patterns during the second nymph stage (Fig 1C and 1D). The miR-71 expression levels showed the opposite wave-like pattern of miR-263 expression levels, with the highest level occurring at the mid-stage for miR-71, whereas miR-263 expression decreased to the lowest level (Fig 1D). In contrast, the mRNA expression of *LmCHS1* was down-regulated at the mid-stage and up-regulated at the early and late stages. However, the mRNA expression of *LmCHT10* was suppressed at the early and late stages and was promoted at the mid-stage (Fig 1C). These data indicate that miR-71/miR263 expression is negatively correlated with *LmCHS1* and *LmCHT10* expression during new integument formation in the nymph stages. The results imply that there is a possible regulatory relationship between the miRNAs and the genes.

### miR-71 and miR-263 regulate *LmCHS1* and *LmCHT10*, respectively

**Fig 2:**
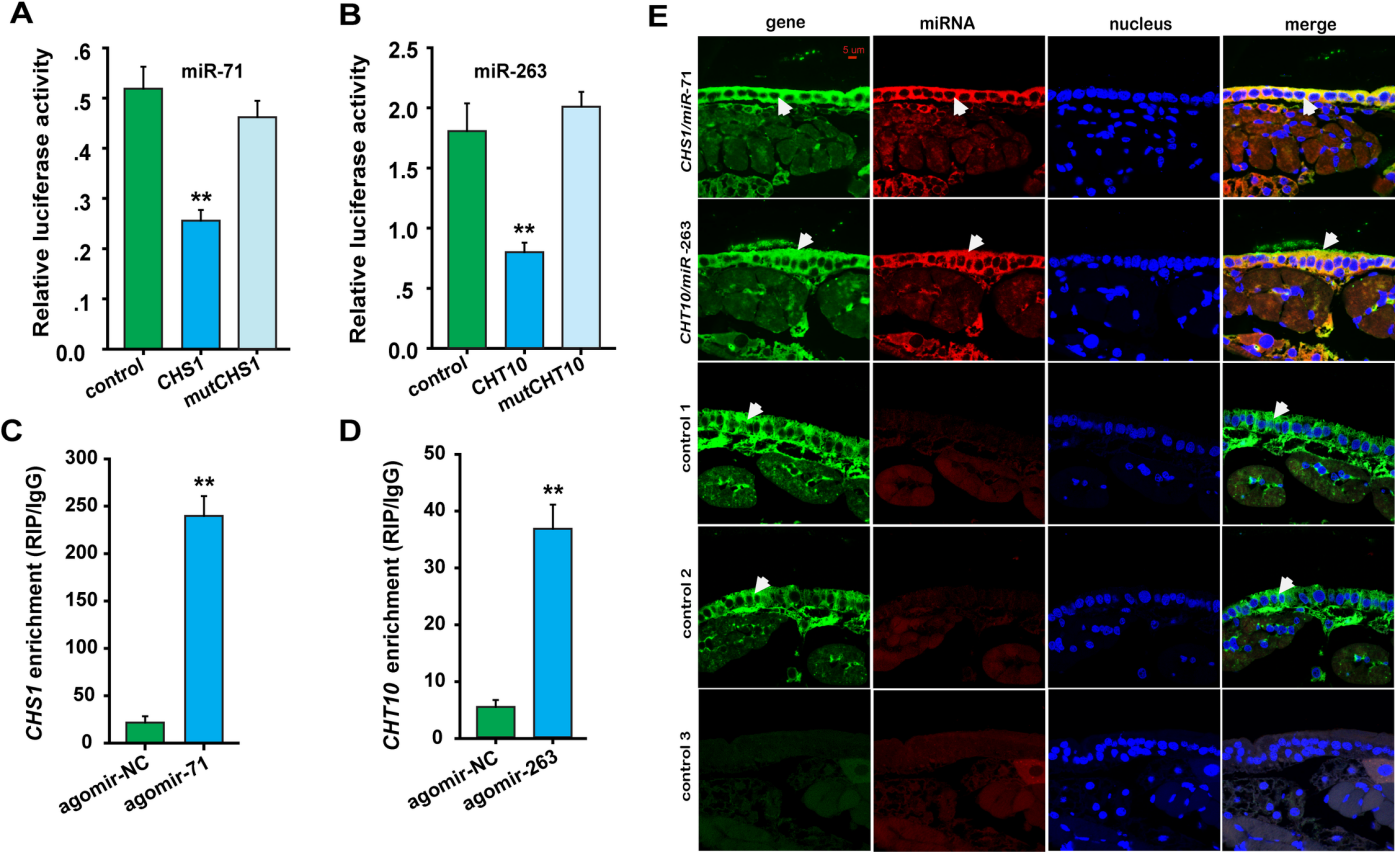
Interactions between miRNAs (miR-71 and miR-263) and their targets *CHS1* and *CHT10* in locusts. (A, B) Luciferase reporter assays were analyzed in S2 cells co-transfected with miR-71 or miR-263 overexpression vectors and psi-CHECK2 vectors containing wild-type (WT) or mutant (MT, the 8 nt of the region that corresponds to the miRNA seed mutated) target gene sequences of *CHS1* (A) and *CHT10* (B) (*n* = 6). (C, D) RIP was performed with an anti-Ago-1 antibody. qPCR analysis was performed to amplify the *CHS1* and *CHT10* mRNAs from the Ago-1 immunoprecipitates from extracts of integument tissue treated with agomir-71 or agomir-263 48 h later compared with the agomir controls (agomir-NC). The data for the luciferase activities and qPCR analyses are presented as means ± SEM (*n* = 6). *p < 0.05; **p < 0.01. (E) miRNAs and their target genes were co-labeled to determine the co-localization between these molecules in the locust integument on the third day and the fifth day of the 2^nd^ instar stage by FISH. Where green (*CHS1* and *CHT10*) and red signals (miR-71 and miR-263) overlap, a yellow signal is observed, indicating the co-localization of miRNAs and their targets. Control 1, scrambled miRNA and *CHS1* antisense probe; Control 2, scrambled miRNA and *CHT10* antisense probe; Control 3, scrambled miRNA, *CHS1* and *CHT10* sense probes. The images were visualized using an LSM 710 confocal fluorescence microscope (Zeiss) at a magnification of 40X.

To confirm the interactions of miR-71, miR-263 and their targeting genes *in vitro*, we performed reporter assays using luciferase constructs fused to the coding region of *LmCHS1* and the 3’UTR of *LmCHT10*. Compared with the agomir control (agomir-NC), the constructs with either the *LmCHS1* or *LmCHT10* binding sites produced lower luciferase activity when co-transfected with miR-71 or miR-263 agomir, respectively, in S2 cells (Fig 2A and 2B). When the regions homologous to the “seed” sequence of miR-71 and miR-263 were mutated in the *LmCHS1* and *LmCHT10* reporter constructs, the luciferase activity returned to levels similar to those produced by mock transfection with the empty reporter plasmid (Fig 2A and 2B). However, the luciferase activity of sites transfected with miR-252, whose expression is negatively correlated with *LmCHS1*, showed no change compared with the control (S3 Fig). To further validate the effect of endogenous miR-71 and miR-263 in S2 cells on the luciferase activity, we investigated miRNA-71 and miR-263 levels in S2 cells. The mir-71 homolog was not detected in the fly genome [36]. The small RNA transcriptome data indicated that only a few reads for miR-263 (7 counts in GEO accession GSM272651 and 1 count in GEO accession GSM272652) were detected in S2 cells, implying a limited expression of miR-263 in S2 cells. We examined luciferase activity in S2 cells with antagomir-263. The luciferase signals of the *CHT10* construct incubated with antagomir-263 did not vary significantly compared with those of the control (S4 Fig). The data suggested that the endogenously expressed miR-263 did not affect the luciferase assay results for the locust miR-263. Thus, the predicted miRNA binding sites in *LmCHS1* and *LmCHT10* are functional and might be targeted by miR-71 and miR-263, respectively, in S2 cells.

Ago1, as a RNA binding protein, is a core component of RISC involved in miRNA-mediated gene silencing. Anti-Ago1 RIP is a biochemical approach to identify the composition and organization of endogenous mRNAs, miRNAs associated with Ago1 proteins. This approach is widely used in interaction validation between miRNA and its target *in vivo*. We then performed an RNA immunoprecipitation assay in the integument to examine the interactions of miR-71 and miR-263 with their targeting genes *in vivo* (Fig 2C and 2D). *LmCHS1* or *LmCHT10* were significantly enriched in the Ago1-immunoprecipitated RNAs from the integuments treated with agomir-71 or agomir-263 compared with those treated with agomir-NC. These results indicated that miR-71 and miR-263 directly regulate *LmCHS1* and *LmCHT10* in the locust integument, respectively.

### Co-localization of the miRNAs and targeting genes

To determine whether miR-71/miR-263 were co-localized in the locust integument, we performed *in situ* analyses of miRNA-71/miR-263 and their targets by miRNA/mRNA fluorescence *in situ* hybridization (FISH). Indeed, we found that miR-71 and *LmCHS1* as well as miR-263 and *LmCHT10* were both widely detected in the epidermal cells of the locust integument (Fig 2E). Specifically, miR-71 is co-localized with *LmCHS1* and miR-263 is co-localized with *LmCHT10* in cells of the integument. The results suggest that in the locust integument, *LmCHS1* and *LmCHT10* interact directly with miR-71 and miR-263, respectively, in a spatial manner.

### miR-71 controls chitin synthesis by targeting *LmCHS1*, and miR-263 inhibits chitin degradation by targeting *LmCHT10*

**Fig 3:**
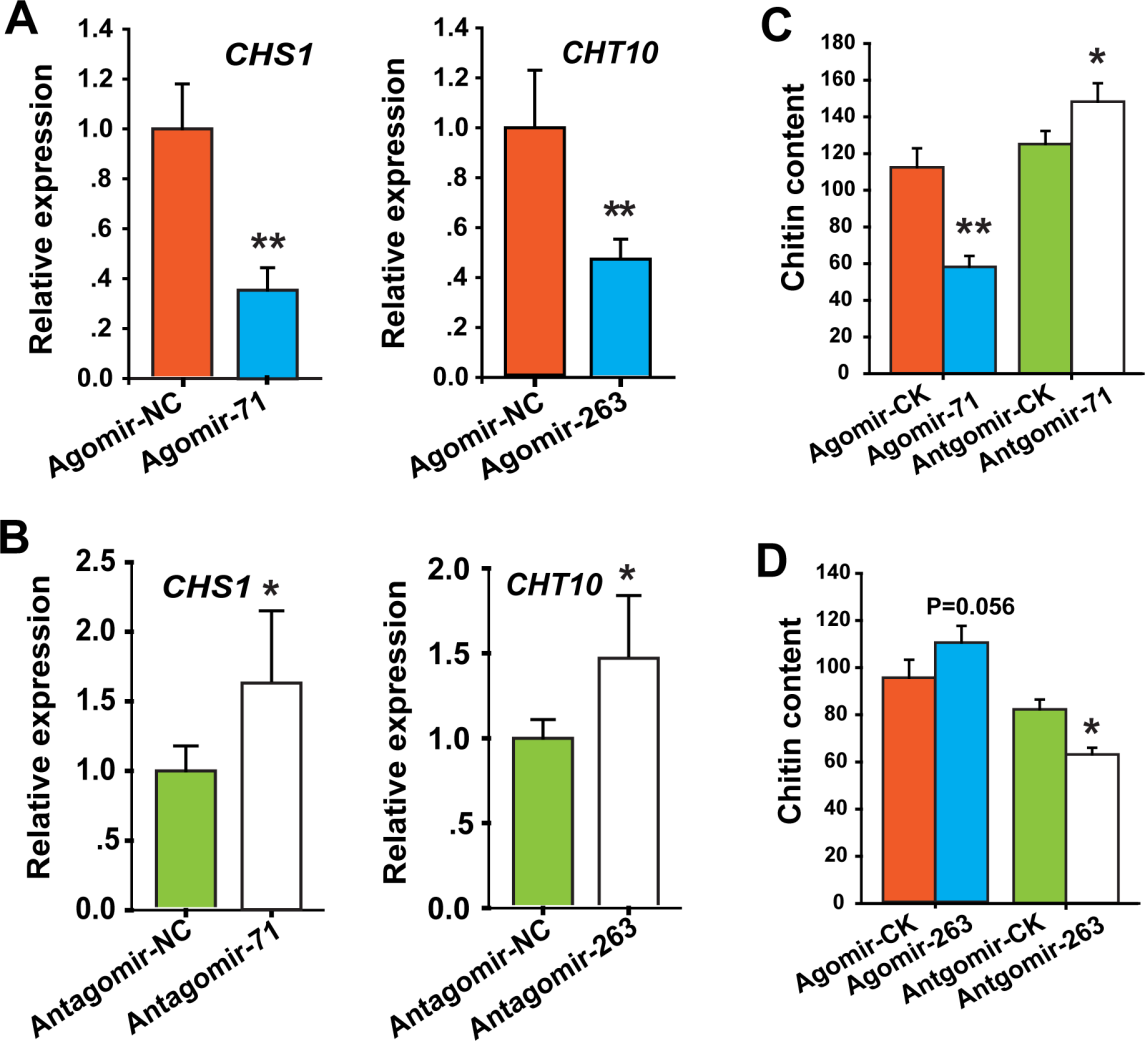
Effect of miR-71 and miR-263 administration on *CHS1* and *CHT10* expression and chitin content in the locust integument. (A, B) The effects of 210 pmol of agomir- and antagomir-71 or agomir- and antagomir-263 treatment for 48 h after injection on the mRNA expression levels of *CHS1* and *CHT10* in locust integuments were studied using qPCR. (C, D) Chitin production was evaluated in the integuments of locusts after treatment with 210 pmol agomir- or antagomir-71/agomir- or antagomir-263 treatment. The qPCR and chitin content data are presented as means ± SEM (*n* = 6). *p < 0.05; **p < 0.01.

To determine the effects of miR-71 and miR263 on their target genes *in vivo*, we detected the expression levels of *LmCHS1* and *LmCHT10* after miRNA agomir (overexpression) or antagomir (knockdown) administration in the locust integument. We first assessed the miRNA expression changes by injecting locusts with miRNA agomir or antagomir *in vivo* to confirm the delivery efficiency of miRNA administration. The qPCR results showed that miR-71 and miR-263 levels were significantly induced and depleted by their agomir and antagomir treatments, respectively. As expected, the treatment had no effect on the expression of the negative control, let-7 (S5 Fig). Moreover, the mRNA levels of *LmCHS1* decreased by approximately 60% compared to those in the control locusts after agomir-71 injection (Fig 3A). Additionally, antagomir-71 injection resulted in a significant up-regulation of the mRNA expression level of *LmCHS1*(Fig 3B). In contrast, inhibition of approximately 55% of *LmCHT10* expression was observed after agomir-263 injection. Additionally, the mRNA levels of *LmCHT10* were up-regulated by miR-263 knockdown (Fig 3A and 3B). No significant effects on the expression of paralog genes *LmCHS2* and *LmCHT5* upon miR-71/miR-263 administration were observed, suggesting that the agomir/antagomir injection specifically acted on the target genes (S6 Fig). Additionally, we sought to determine whether the administration of miR-71 had any effects on the expression of miR-263, or vice versa. The results indicated that there was no interaction between miR-71 and miR-263, which were involved in the regulation of two distinct processes (chitin synthesis and degradation) (S7 Fig). Since 20E is believed to primarily regulate insect growth and development processes, including molting [2,37], we further used 20E treatments to investigate whether 20E might play a relevant role in the regulation of the expression of miR-71 and miR-263. The results indicated that 20E treatment depressed the expression levels of miR-263, but did not have significant effects on miR-71 expression level (S8 Fig).

Because *LmCHS1* and *LmCHT10* are essential genes in chitin synthesis and degradation, we determined the effects of miR-71 and miR-263 on chitin production in the integuments after miRNA administration *in vivo*. The administration of the miR-71 agomir reduced chitin production by approximately 48% (Fig 3C). In contrast, miR-71 knockdown significantly increased the chitin content (by approximately 18%, *p* < 0.05) of the locusts. Accordingly, a significant increase in chitin content was observed after agomir-263 manipulation, and antagomir-263 injection decreased chitin content (Fig 3D). Thus, administration of miR-71 and miR-263 may have profound regulatory effects on chitin production and content *in vivo* in locusts.

### miR-71 and miR-263 regulate the molting process of locusts by inhibiting chitin metabolism

**Fig 4:**
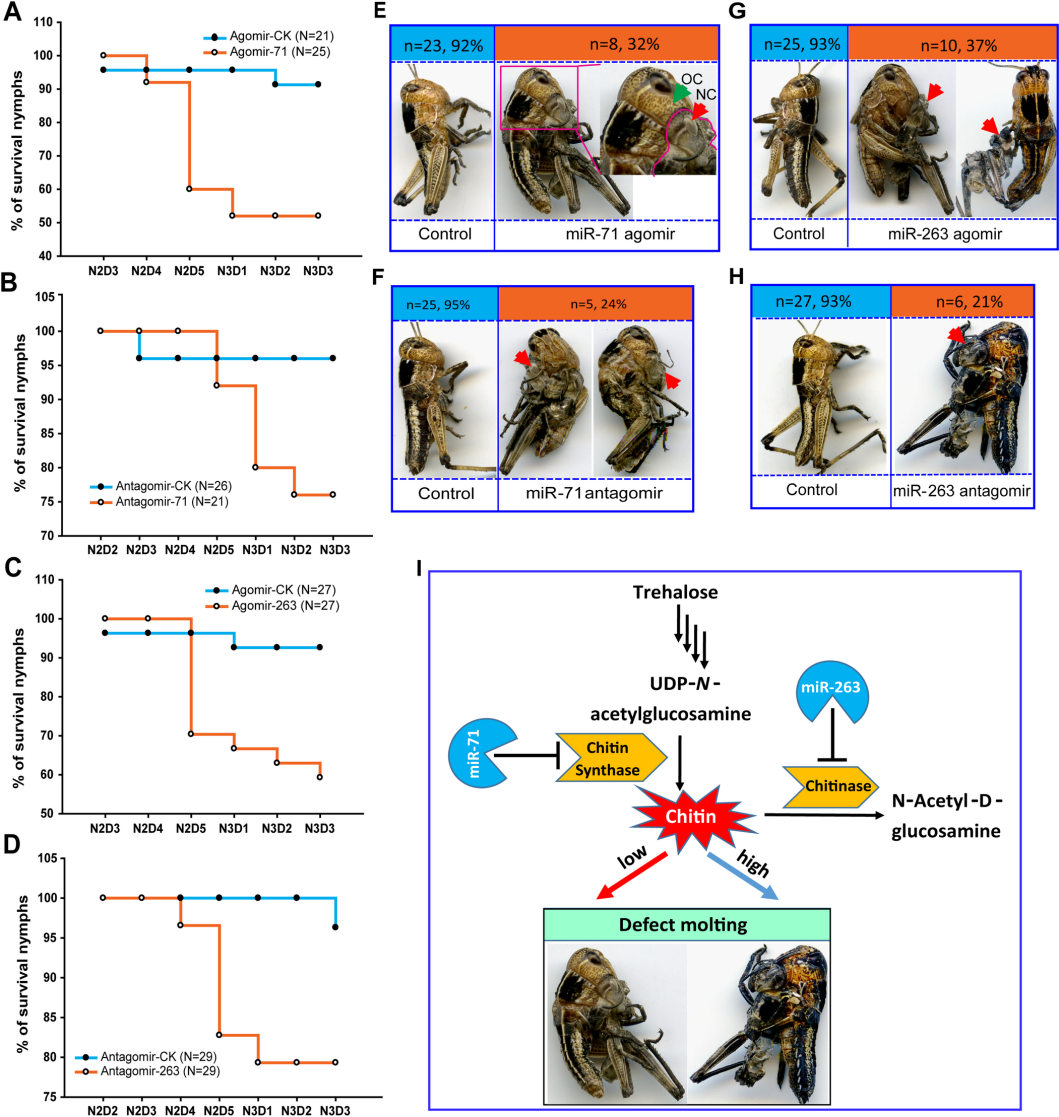
miR-71 and miR-263 hinder molting of the migratory locust. (A-D) The effects of 210 pmol agomir-71 or agomir-263 and antagomir-71 or antagomir-263 treatment on the mortality of locusts were studied after injection. N, the number of animals treated. (E-H) The effects of 210 pmol agomir-71 or agomir-263 and antagomir-71 or antagomir-263 treatment on the molt phenotype of locusts were studied after injection. The red arrow indicates the shedding old cuticle in the defective molting process. OC, old cuticle, NC, new cuticle. (I) Model of the miR-71- and miR-263-mediated chitin metabolism pathway associated with the molting of the migratory locust. miR-71 controls chitin production by regulating *CHS1*, and miR-263 controls chitin degradation by modulating *CHT10* in the locust integument.

To determine the function of miR-71 and miR-263 during the molting process, we monitored the molting of locusts after agomir-71 or agomir-263 injection, respectively. The locusts injected with agomir-71 or agomir-263 displayed a distinct molting defect phenotype. In total, of 25 nymphs injected with agomir-71, 12 (48%) died during the molting process from second instar to third instar, whereas only 8.7% (2 out of 23) of the control nymphs died during this process (Fig 4A). Similarly, after injection of agomir-263, the mortality reached 40.7%, which was significantly higher than that of the control (only 7.4%) during the molting process (Fig 4C). In parallel, miR-71 and miR263 knockdown caused by injection of antagomir-71 or antagomir-263 resulted in incomplete ecdysis, with 24.0% and 20.7% mortality, respectively (Fig 4B and 4D).

After nymphs were injected with the miR-71 and miR-263 agomir or antagomir, the nymphs exhibited abnormal and unsuccessful molting (Fig 4E–4H), in which a certain amount of the old cuticle was separated from the body but not detached from the body to any extent. Moreover, some nymphs showed a molting defect, in which legs failed to slough from the old cuticle, and other nymphs died without obvious molting defects due to molting arrest (Fig 4E–4H).

### The chitin contents of new/old cuticle were regulated by miR-71 and miR-263 in molting

**Fig 5:**
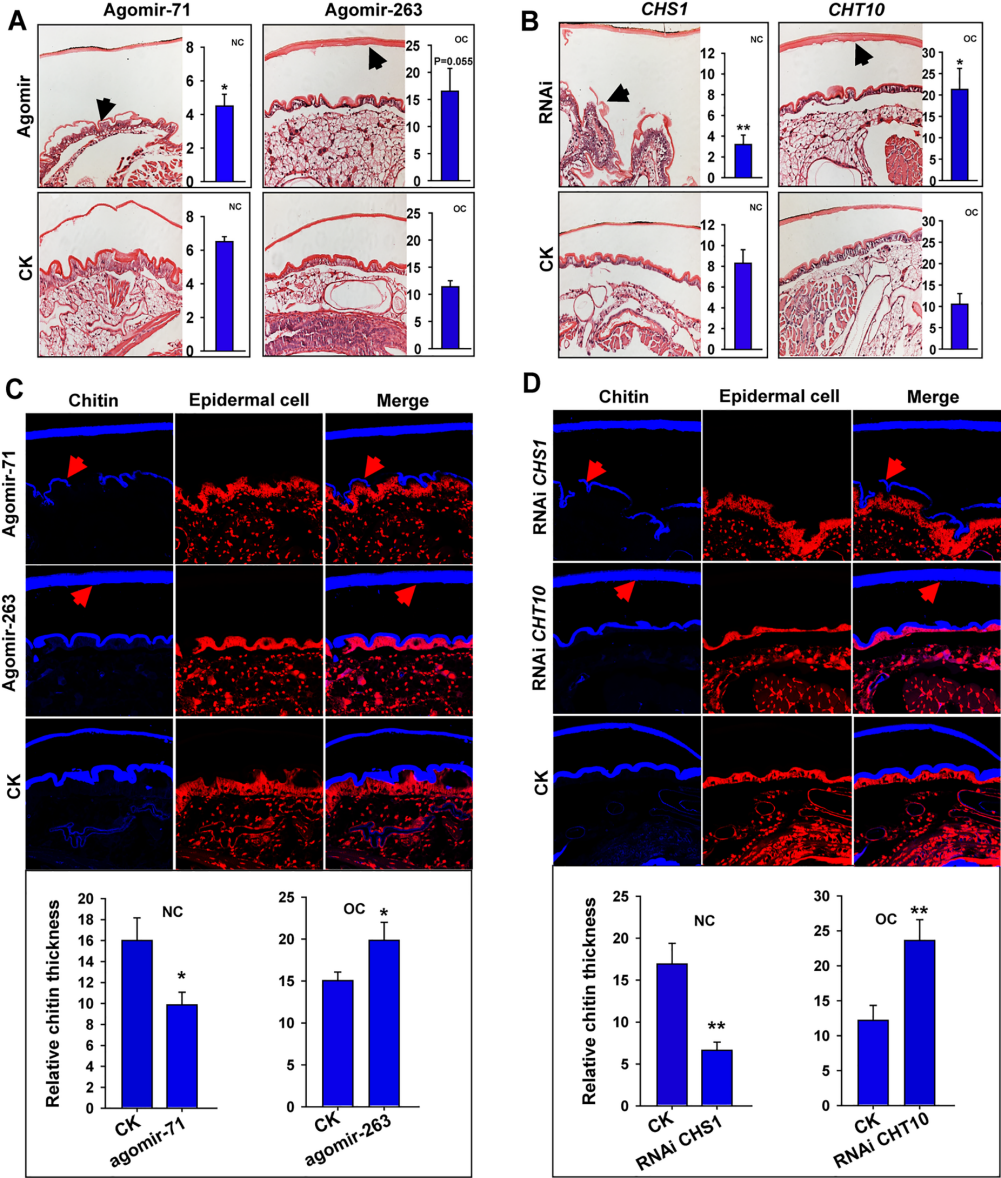
Effect of miR-71 and miR-263 administration on the chitin metabolism of the new and old cuticle. (A, B) Hematoxylin and eosin staining of the integument was performed by injecting agomir-71/agomir-263 (A) or by injecting dsRNA against *CHS1*/*CHT10* (B) into the locusts. (C, D) The chitin staining experiment in the integument was performed by injecting agomir-71/-263 (C) or injecting dsRNA against *CHS1*/*CHT10* (D) into the locusts. The chitin and cuticle thicknesses of the new cuticle/old cuticle were quantified using ZEN 2.1 software and expressed as means ± SEM (*n* = 4). OC, old cuticle, NC, new cuticle. *p < 0.05; **p < 0.01.

Treatment of locusts with agomir-71 and agomir-263 resulted in the down-regulation of *LmCHS1* and *LmCHT10* expression and a corresponding change in chitin content, thereby generating a significant molting defect (Fig 4I). To further determine whether the abnormal layer of the cuticle was responsible for the molting defect induced by miR-71 and miR-263, we performed hematoxylin and eosin staining and chitin staining in the integument by injecting agomir-71 or agomir-263 into the locusts (Fig 5A and 5C). A significant decrease in chitin content occurred in response to miR-71 overexpression in the newly formed cuticle, which exhibited a severe deficiency due to diminished chitin synthesis (Fig 5A). Similarly, RNAi for *LmCHS1* prevented the synthesis of cuticle chitin, as expected in the newly formed cuticle (Fig 5B and 5D). Conversely, miR-263 overexpression inhibited the degradation of the old cuticle, the chitin of which was not diminished compared with that of the agomir controls (ig 5A and 5C). Consistent with the miR-263-mediated phenotype, knockdown of *LmCHT10* transcripts hindered chitin degradation of the old cuticle, leading to a dramatically thickened layer of the old cuticle and impeding the shedding of the old cuticle during molting (Fig 5B and 5D). Therefore, the change in chitin content of new/old cuticle regulated by miR-71 and miR-263 is a key mediator of defective molting.

## Discussion

Our previous studies confirmed that depletion of Dicer-1 prevented the molting process [38] and chitin-metabolic genes, including *LmCHS* and *LmCHT*, are related to molting in the migratory locust [7]. The results presented herein explored the link between the Dicer-mediated phenotype and *LmCHS*/*LmCHT*-associated molting. In this study, we found that miR-71 and miR-263 control chitin synthesis and degradation by targeting *LmCHS1* and *LmCHT10*, resulting in post-transcriptional regulation of the molting process in locusts (Fig 4I). This miRNA-mediated mechanism of chitin metabolism provides insight into the molecular basis of the molting process in locusts.

We found that miR-71 targets *LmCHS1* and miR-263 targets *LmCHT10* in the chitin metabolic pathway of locusts. Chitin synthases and chitinases are responsible for the synthesis or degradation of chitins, which represent two inverse processes [39]. Thus, we suspected that the two processes of chitin synthesis and degradation affect one another. Therefore, we tested whether these two miRNAs interacted with each other using agomir-71/263 treatment. However, this was not the case (S7 Fig). The expression levels of the two miRNAs were very similar to those of the respective controls. In addition, 20-hydroxyecdysone (20E), as a key steroid hormone, coordinates multiple developmental events involving insect molting and metamorphosis [40]. Although 20E treatment is considered to be correlated with *CHS* and *CHT* expression, its roles in the regulation of *CHS* and *CHT* remain a matter of controversy. *DmeCHS-1* and *DmeCHS-2* transcription is activated by 20E during *Drosophila* metamorphosis [41]. *MsCHS-1* gene is negatively controlled by 20E, reflecting a dual effect of 20E [42]. *LmCHT5* and *TmCHT5* gene expression can be induced by 20E during the molting process [43,44]. However, the pathway linking 20E to *CHS* or *CHT* is still largely unknown. miR-8-5p and miR-2a-3p act as molecular regulators that tune the chitin biosynthesis pathway in response to 20E [45]. Thereby, we wondered whether 20E might play a role in the regulation of the expression of miR-71 and miR-263, leading to the precise expression of *LmCHS1* and *LmCHT10*. The 20E treatment inhibited miR-263 expression and induced *LmCHT10* expression but did not affect the expression levels of miR-71 or *LmCHS1* (S1 Fig). Thus, the 20E-miR-263-*CHT10* axis may switch the degradation of chitin on and off, whereas miR-71-*CHS1* axis-mediated chitin synthesis is regulated by mechanisms other than 20E.

The regulatory function of miR-263 is conserved across a broad range of insect species. We performed miRNA target prediction in other insect species for which *CHT10*/*CHS1* UTR sequences were available in the NCBI GenBank. The prediction results revealed that miRNA-263 binding sites of *CHT10* are present in several holometabolous insects including flies, beetles and mosquitoes, whereas no binding sites for miR-71 were identified in other insect species (S3 Table). This finding suggests that these regulatory interactions have been evolutionarily conserved, indicating that there is selective pressure to maintain the regulatory interactions of miR-263 and *CHT10* across species. Previous studies have shown that the expression patterns of *CHT10* during developmental instar stages are quite similar among insects [15,46], suggesting a common regulatory mechanism of the miRNA-263-dependent molting process. Our study provided experimental evidence that this regulatory mechanism is also present in hemimetabolous locusts. Members of Orthoptera occupy a more basal position in the insect lineage relative to holometabolous insects [47,48]. Thus, we presume that the regulatory roles of miR-263 in the molting process represent an ancestral function in insects that perhaps originated with the emergence of the common ancestor of hemimetabolous and holometabolous insects.

miRNAs are precise regulators that are able to sharpen developmental transcription by increasing and reducing target expression to meet developmental demands [49]. Due to the reduced expression of *CHS1* and *CHT10*, the agomir treatments of miRNA-71 and miR-263 can cause severe deficiency of new cuticle synthesis and failed degradation of the old cuticle, respectively. Unexpectedly, the antagomir treatments of miR-71/miR-263 and the resulting up-regulation of their target genes resulted in a similar aberrant phenotype, indicating that the elevated ectopic expression of *CHS1*/*CHT10* is also detrimental to the molting process. These results indicate that extremely high or low expression of *CHS1* and *CHT10* during a critical period of the molting process can result in the development of an aberrant molting phenotype. miRNAs could tune the transcriptional activities of target genes to physiologically relevant levels [50]. miR-71/miR-263 could directly interact with *CHS1*/*CHT10* and play a role in ensuring an accurate level of their expression. The precise interactions of miR-71/miR-263 and *CHS1*/*CHT10* regulate the molting process in a spatio-temporal manner. Taken together, our results show that the transcriptional activities of *CHS1* and *CHT10* are tuned to a precise level at which they can execute proper function, emphasizing the important roles of miRNA-mediated precise regulation in the molting process.

The conclusion that emerges is that two miRNAs control the molting process by precisely regulating chitin metabolism. miR-71 and miR-263 suppress *CHS1* and *CHT10* transcript levels, thus preventing the progression of the molt to the next stage. This miRNA-mediated post-transcriptional regulation of chitin metabolism is particularly significant for understanding the molting process of locusts and potentially provides new targets for controlling locust plagues worldwide.

## Materials and Methods

### Insects

Locusts were obtained from the same locust colonies, which were maintained at the Institute of Zoology, Chinese Academy of Sciences, China. Nymphs were reared under a 14:10 light/dark photo regime at 30 ± 2°C and were fed fresh wheat seedlings and bran.

### Small RNA transcriptome sequencing

Total RNA was extracted using TRIzol (Invitrogen) and treated with DNase I following the manufacturer’s instructions. The RNA concentration and purity were assessed in an Agilent 2100 Bioanalyzer (Agilent) to verify RNA integrity. Small RNA libraries were constructed using a TruSeq small RNA sample preparation kit (Illumina). Briefly, the 3’ and 5’ RNA adapters were ligated to the corresponding ends of small RNAs. Following adapter ligation, the ligated RNA fragments were reverse transcribed using M-MLV reverse transcriptase (Invitrogen). The resulting cDNA products were PCR amplified with two primers that were complementary to the ends of the adapter sequences. The PCR amplicons were separated by size in 6% Novex polyacrylamide gel for miRNA enrichment and sequenced on an Illumina Genome Analyzer IIx sequencing system. Using the Cutadapt software, we trimmed the low-quality reads and the reads showing sequence similarity to adaptor sequences at the start or end terminals. The quantifier module in the miRDeep (version 2.0.0.5) software package was used to measure expression levels based on read counts.

### *In vitro* luciferase validation

The ~300-bp sequences of the CDS and the 3′ UTR surrounding the predicted miR-71 and miR-263 target sites in *CHS1* and *CHT10*, respectively, were separately cloned into the psiCHECK-2 vector (Promega) using the XhoI and NotI sites. To generate the mutation version, the 8 nt of binding sites were mutated (GTTTTTCA for *CHS1*; GTGCTATT for *CHT10*), which include the region complementary to the miR-71 and miR-263 seed. S2 cells were co-transfected with 800 ng of the luciferase reporter vector or the empty vector and agomir-71 (-263) at a 1:4 ratio using the Lipofectamine™ 2000 reagent (Invitrogen) according to the manufacturer’s instructions. The activities of the firefly and *Renilla* luciferases were measured 48 h after transfection with the Dual-Glo Luciferase Assay System (Promega) using a luminometer (Promega). Results are expressed as the ratio Renilla/firefly luciferase activity (mean ± SEM) based on six independent replicates.

### miRNA agomir and antagomir treatment *in vivo*

The miRNA agomir or antagomir, each of which is a stable miRNA mimic or inhibitor, was used to validate the function of the miRNA *in vivo*. Briefly, 210 pmol of agomir-71 (-263) or antagomir-71 (-263) (500 μM; RiboBio) was injected into the thoracic hemocoels of second-stadium nymphs two times at 48 h intervals. The agomir or antagomir negative controls (500 μM) were also injected into the locust thoracic hemocoels (RiboBio). All injections were administered using a nanoliter injector (World Precision Instruments) with a glass micropipette tip. Treated nymphs were subjected to phenotypic observation of molting process. Their integuments were harvested, snap-frozen, and stored at -80°C.

### Assays of quantitative PCR for mRNA and miRNA

Total RNA enriched for small RNAs was isolated from integuments using the mirVana miRNA isolation kit (Ambion). Moloney murine leukemia virus (M-MLV) reverse transcriptase (Promega) and a miRNA first-strand cDNA synthesis kit (Ambion) were used to prepare the Oligo (dT)-primed cDNA and stem-loop cDNA, respectively. The miRNAs and mRNAs were subjected to qPCR using the SYBR Green miRNA expression and gene expression assays, respectively, according to the manufacturer’s instructions (Tiangen); qPCR was performed on a LightCycler^®^ 480 instrument (Roche). The PCR data were analyzed using the 2^−ΔΔCt^ method of relative quantification. As endogenous controls, U6 snRNA and ribosomal protein *RP49* were used to quantify the miRNA and mRNA expression levels, respectively. Dissociation curves were determined for each miRNA and mRNA to confirm unique amplification. The qPCR primers are listed in S4 Table. All the qRT-PCR reactions were performed in six biological replicates. Four integuments were involved in one biological replicate.

### Co-localization of miRNA and its targets by fluorescence *in situ* hybridization

A combined two-color fluorescence *in situ* analysis of miRNA-71 (-263) and its targets was performed on the integuments of second-instar nymphs by co-labeling of the miRNA and its target according to the method described by Nuovo et al. [51]. An antisense locked nucleic acid (LNA) detection probe for miR-71, miR-263 or a scrambled control (Exiqon) was labeled with double digoxigenin. Biotin-labeled antisense and sense probes of *CHS1* and *CHT10* were generated from linearized recombinant pGEM-T Easy plasmids using the T7/SP6 RNA transcription system (Roche, Basel, Switzerland) following the recommended protocols. Based on the timepoint at which higher expression activities were observed for miR-71 and miR-263, we selected the nymphs on the third day of the 2^nd^ instar stage for miR-71 and *CHS1* co-localization detection and the nymphs on the fifth day of the 2^nd^ instar stage for miR-263 and *CHT10* co-localisation detection, respectively. The integuments were fixed in 4% paraformaldehyde overnight. The paraffin-embedded integument tissue slides (5 μm thick) were deparaffinized in xylene, rehydrated with an ethanol gradient, digested with 20 μg/mL proteinase K (Roche) at 37°C for 15 min, and incubated with the LNA miRNA probes and its target RNA probe at 60°C for 5 min. The slides were then hybridized for 7–15 h at 37°C and washed in 0.2× SSC and 2% BSA at 4°C for 5 min. The slides were incubated in anti-digoxigenin–alkaline phosphatase conjugate (1:150 dilution) for 30 min at 37°C, followed by incubation with the HNPP substrate. For biotin-labeled probes, a TSA kit (Perkin Elmer, MA, USA) including a streptavidin horse radish peroxidase-conjugate and fluorescein tyramide substrate was used. The signals of the miRNA and its target were detected using an LSM 710 confocal fluorescence microscope (Zeiss). The primes for probe synthesis of *CHS1* and *CHT10* are listed in S4 Table.

### RNA immunoprecipitation (RIP) assays

The RIP assay was performed using a Magna RIP Quad kit (Millipore) according to the manufacturer’s instructions, with slight modifications. The 2-day-old second instar nymphs were microinjected with agomir-71 or agomir-263. A scrambled miRNA agomir was used as a negative control. Treated nymphs were subjected to RIP analysis 48 h later. Eight integuments of abdomen were collected and homogenized in ice-cold RIP lysis buffer. The homogenates were stored at -80°C overnight. A total of 5 μg of Ago-1 antibody (Abmart) or normal mouse IgG (Millipore), which was used as a negative control, was pre-incubated with magnetic beads. The frozen homogenates in the RIP lysates were thawed and centrifuged, and the supernatants were incubated with the magnetic bead–antibody complex at 4°C overnight. The immunoprecipitated RNAs were reverse-transcribed into cDNA using random hexamers. qPCR was performed to quantify *LmCHS1* and *LmCHT10*. The supernatants of the RIP lysates (input) and the IgG controls were assayed to normalize the relative expression levels of the target genes.

### Quantification of chitin in the integument tissue extracts

The chitin content of the locust integuments was quantified after miR-71 or miR-263 administration. The integument tissues of the locust nymphs were immediately dissected and stored in liquid nitrogen. Three integuments of locust abdomens were homogenized in liquid nitrogen and transferred to 3% SDS. The homogenates were incubated at 100°C for 15 min; then, 120% KOH was added. The pellets were re-suspended and incubated at 130°C for 1 h. After cooling, 0.8 ml ice-cold 75% ethanol was added, and the samples were shaken until the KOH and ethanol formed a single phase. The homogenates were then centrifuged at 4°C, and the supernatants were discarded. The pellets were washed with 40% cold ethanol containing insoluble chitosan. Approximately 50 μl of 10% NaNO_2_ and 50 μl of 10% KHSO_4_ were added to each sample, and the samples were centrifuged at 4°C. The supernatants were combined with 20 μl of 12.5% NH_4_SO_3_NH_2_ and 20 μl freshly prepared 0.5% (wt/vol) 3-methyl-2-benzothiazolone hydrazone hydrochloride hydrate solution, and the reaction was heated to 99.9°C for 3 min. After cooling, 20 μl of 0.83% FeCl_3_.6H_2_O solution was added to the reaction. Measurements of the reaction mixture were performed using a microplate reader at 650 nm using glucosamine as a standard.

### RNA interference

To knock down *CHS1* and *CHT10*, double-stranded RNA (dsRNA) was synthesized using T7 RiboMAXTM Express RNAi System (Promega, USA) following the manufacturer’s instructions. Each insect was injected with 3 μg of dsRNAs at day 3 of the second instar nymphs. Control nymphs were injected with equivalent volumes of dsGFP alone. Total 25 nymphs were injected with dsRNA for each gene. Nymphs were observed carefully after injection. The nymphs that typically showed abnormal ecdysis were used for subsequent extraction. Six abnormal nymphs and six control nymphs were used for hematoxylin and eosin staining and chitin staining.

### Statistical analysis

The SPSS 17.0 software (SPSS Inc.) was used for statistical analysis. The differences between treatments were compared using either Student’s *t*-test or one-way analysis of variance (ANOVA) followed by Tukey’s test for multiple comparisons. The Mann–Whitney *U* test was used to analyze the behavioral data due to its non-normal distribution characteristics. *p* < 0.05 was considered statistically significant. All results are expressed as means ± SEM.

## Supporting Information

S1 Fig Target prediction of miR-71 and miR-263 in the *CHS1* and *CHT10* genes of *Locusta migratoria* by RNAhybrid program. (TIF)

S2 Fig Expression of miRNAs containing potential binding sites with *CHS1* and *CHT10* by prediction using miRanda bioinformatics software were determined in the integuments of locusts by qRT-PCR. The data are presented as means ± SEM (*n* = 6).
(TIF)

S3 Fig Validation of miR-252 site not in *CHS1* mRNA of the locust. Luciferase reporter assays were analyzed in S2 cells co-transfected with miR-252 agomir and psi-CHECK2 vectors containing the target gene sequence of *CHS1* (*n* = 6). The data for the luciferase activities are presented as means ± SEM (*n* = 6).
(TIF)

S4 Fig Validation of miR-263 site in *CHT10* mRNA of the locust. Luciferase reporter assays were analyzed in S2 cells co-transfected with miR-263 antagomir and psi-CHECK2 vectors containing target gene sequences of *CHT10* (*n* = 6). The data for the luciferase activities are presented as means ± SEM (*n* = 6).
(TIF)

S5 Fig Effect of miR-71/miR-263 overexpression or silencing on the expression of miR-71/miR-263. (A, B) The expression levels of miR-71 and miR-263 were determined 48 h after treatment with 210 pmol agomir-71/ agomir-263 (A) or antagomir-71/antagomir-263 (B) using qPCR respectively. (C) The expression levels of let-7 were determined 48 h after treatment with 210 pmol agomir-71/agomir-263 or antagomir-71/antagomir-263 using qPCR, respectively.
(TIF)

S6 Fig Effect of miR-71/miR-263 overexpression on the expression of other members of their target gene family. (A) *CHS2* expression was quantified using qRT-PCR 48 h after treatment of locusts with 210 pmol agomir-71. (B) *CHT5* expression was quantified using qRT-PCR 48 h after treatment of locusts with 210 pmol agomir-263. The data are presented as means ± SEM (*n* = 6). *p < 0.05; **p < 0.01.
(TIF)

S7 Fig Effect of miR-71/miR-263 overexpression or silencing on the expression of miR-263/miR-71. (A) The expression levels of miR-263 were quantified using qPCR 48 h after the locusts were treated with 210 pmol of agomir- and antagomir-71. (B) The expression levels of miR-71 were quantified using qPCR 48 h after the locusts were treated with 210 pmol of agomir- and antagomir-263. All data are presented as means ± SEM (*n* = 6).
(TIF)

S8 Fig Effect of 20E on the expression levels of miR-71, miR-263 and their targets genes. (A) miR-71 and miR-263 expression was quantified using qRT-PCR 2 h and 6 h after the locusts were treated with 20E. (B) The expression levels of *CHS1* and *CHT10* were quantified using qRT-PCR 2 h and 6 h after the locusts were treated with 20E. The data are presented as means ± SEM (*n* = 6). **p < 0.01.
(TIF)

S1 Table miRNAs identified in the integument of *Locusta migratoria* that contained the potential binding site with *CHS1* as predicted by the miRanda software. (XLSX)

S2 Table miRNAs identified in the integument of *Locusta migratoria* that contained the potential binding site with *CHT10* as predicted by the miRanda software. (XLSX)

S3 Table miRNAs contained the potential binding site with *CHT10* as predicted by the miRanda software in other species. (XLSX)

S4 Table The primary primers used in the study. (XLS)
