# Supplementary figures and images for: miR-71 and miR-263 Jointly Regulate Target Genes Chitin synthase and Chitinase to Control Locust Molting
Source: PLoS Genet. 2016 Aug 17;12(8):e1006257. doi: 10.1371/journal.pgen.1006257 (PMC4988631; doi:10.1371/journal.pgen.1006257)

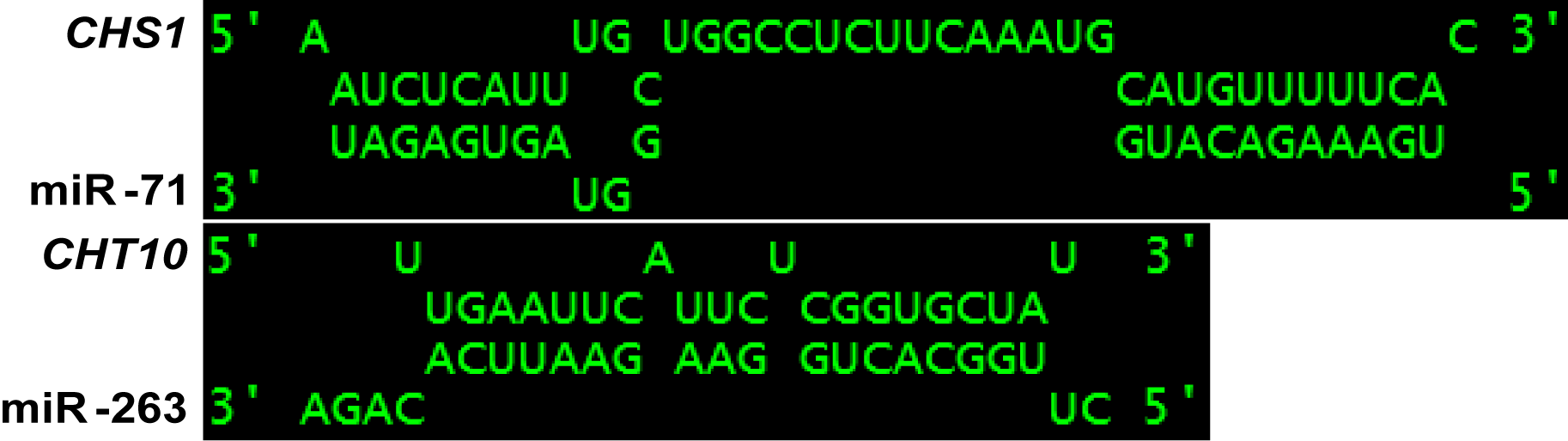

Supplement: S1 Fig — (TIF) [file pgen.1006257.s001.tif]

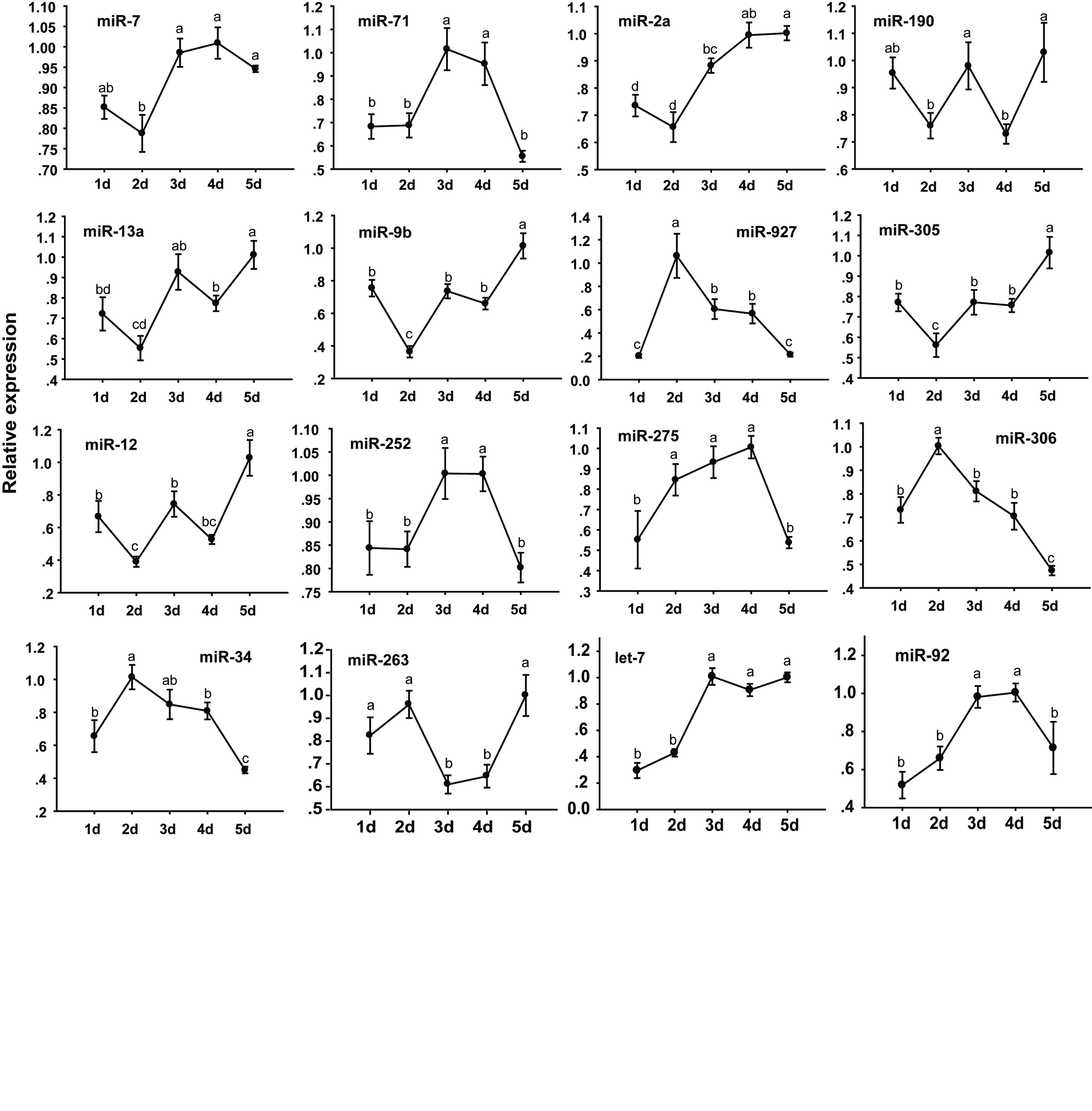

Supplement: S2 Fig — The data are presented as means ± SEM (n = 6). (TIF) [file pgen.1006257.s002.tif]

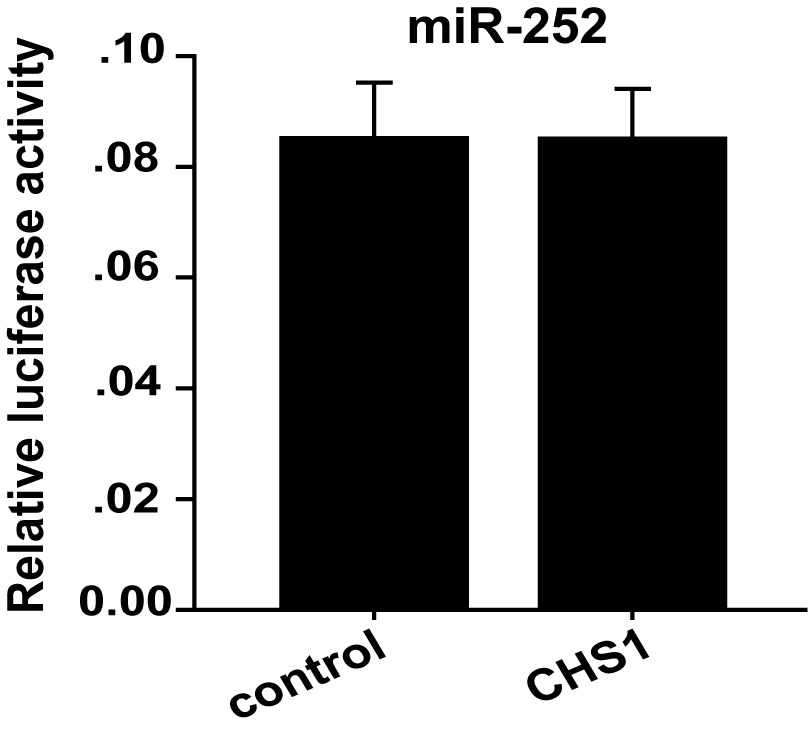

Supplement: S3 Fig — Luciferase reporter assays were analyzed in S2 cells co-transfected with miR-252 agomir and psi-CHECK2 vectors containing the target gene sequence of CHS1 (n = 6). The data for the luciferase activities are presented as means ± SEM (n = 6). (TIF) [file pgen.1006257.s003.tif]

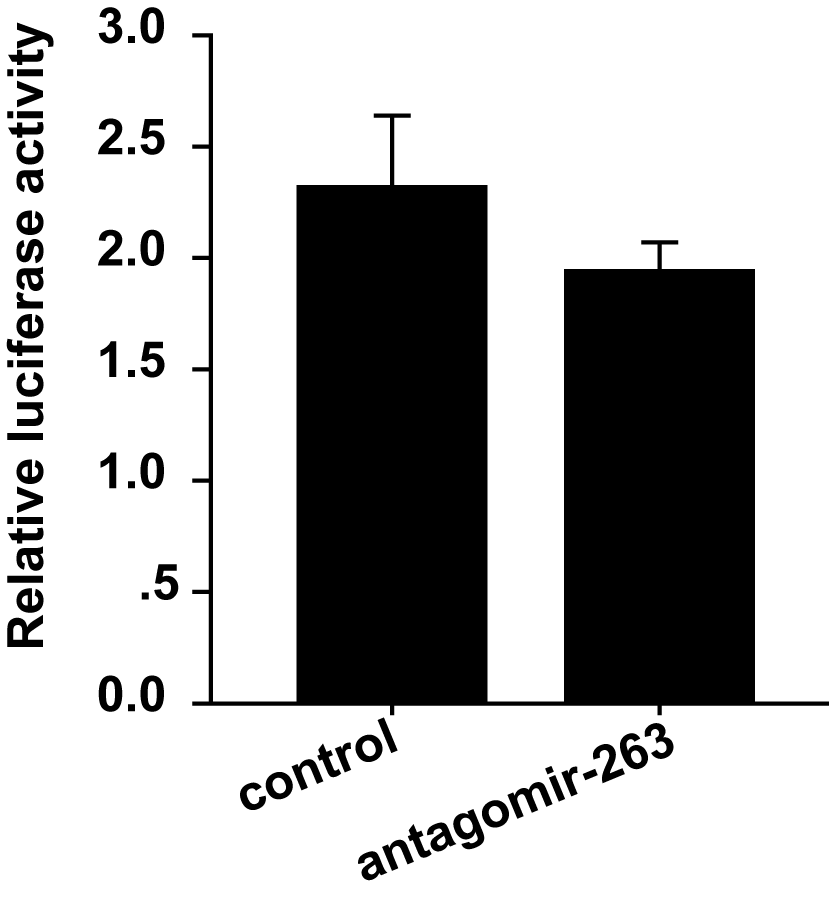

Supplement: S4 Fig — Luciferase reporter assays were analyzed in S2 cells co-transfected with miR-263 antagomir and psi-CHECK2 vectors containing target gene sequences of CHT10 (n = 6). The data for the luciferase activities are presented as means ± SEM (n = 6). (TIF) [file pgen.1006257.s004.tif]

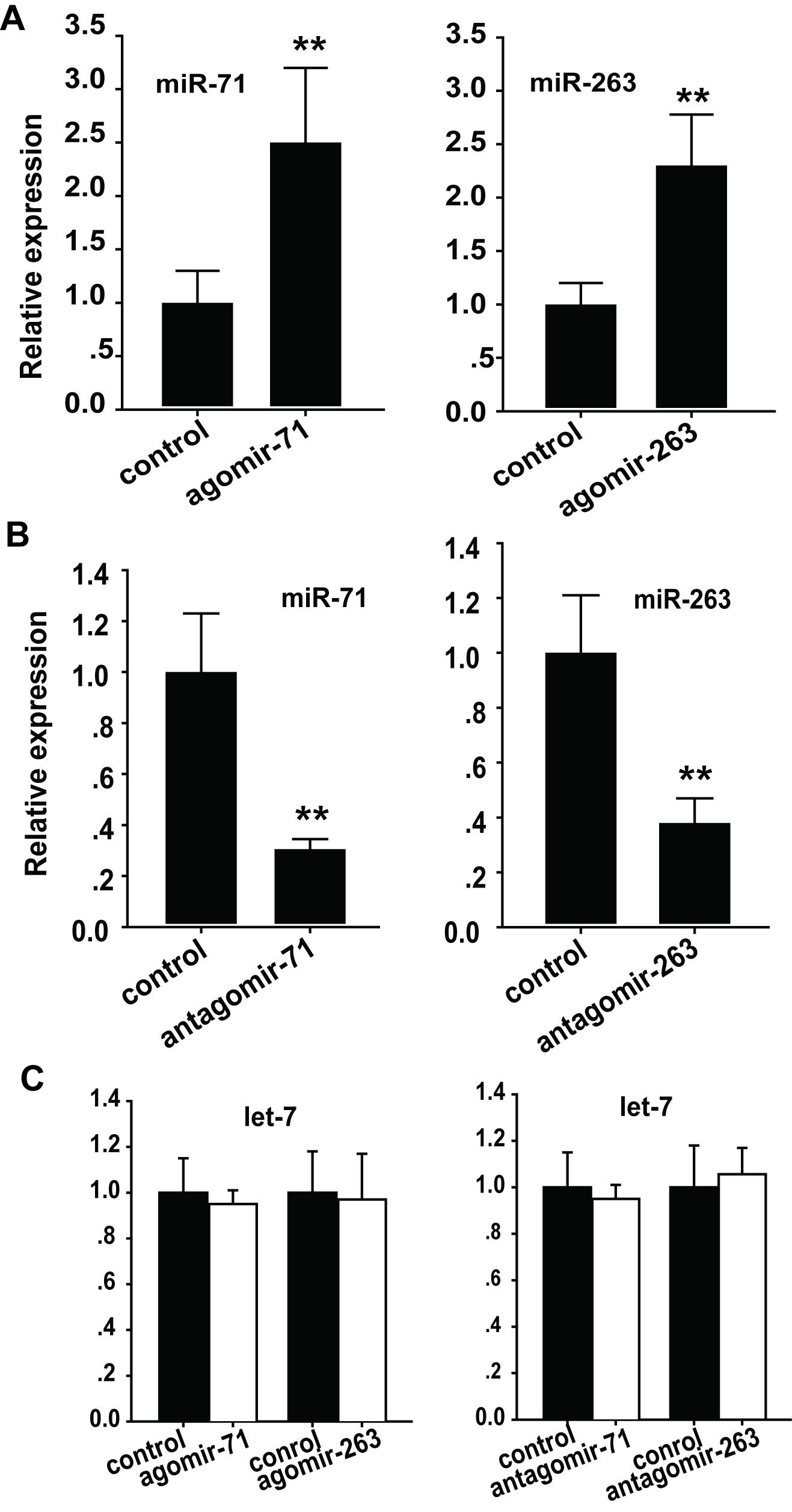

Supplement: S5 Fig — (A, B) The expression levels of miR-71 and miR-263 were determined 48 h after treatment with 210 pmol agomir-71/ agomir-263 (A) or antagomir-71/antagomir-263 (B) using qPCR respectively. (C) The expression levels of let-7 were determined 48 h after treatment with 210 pmol agomir-71/agomir-263 or antagomir-71/antagomir-263 using qPCR, respectively. (TIF) [file pgen.1006257.s005.tif]

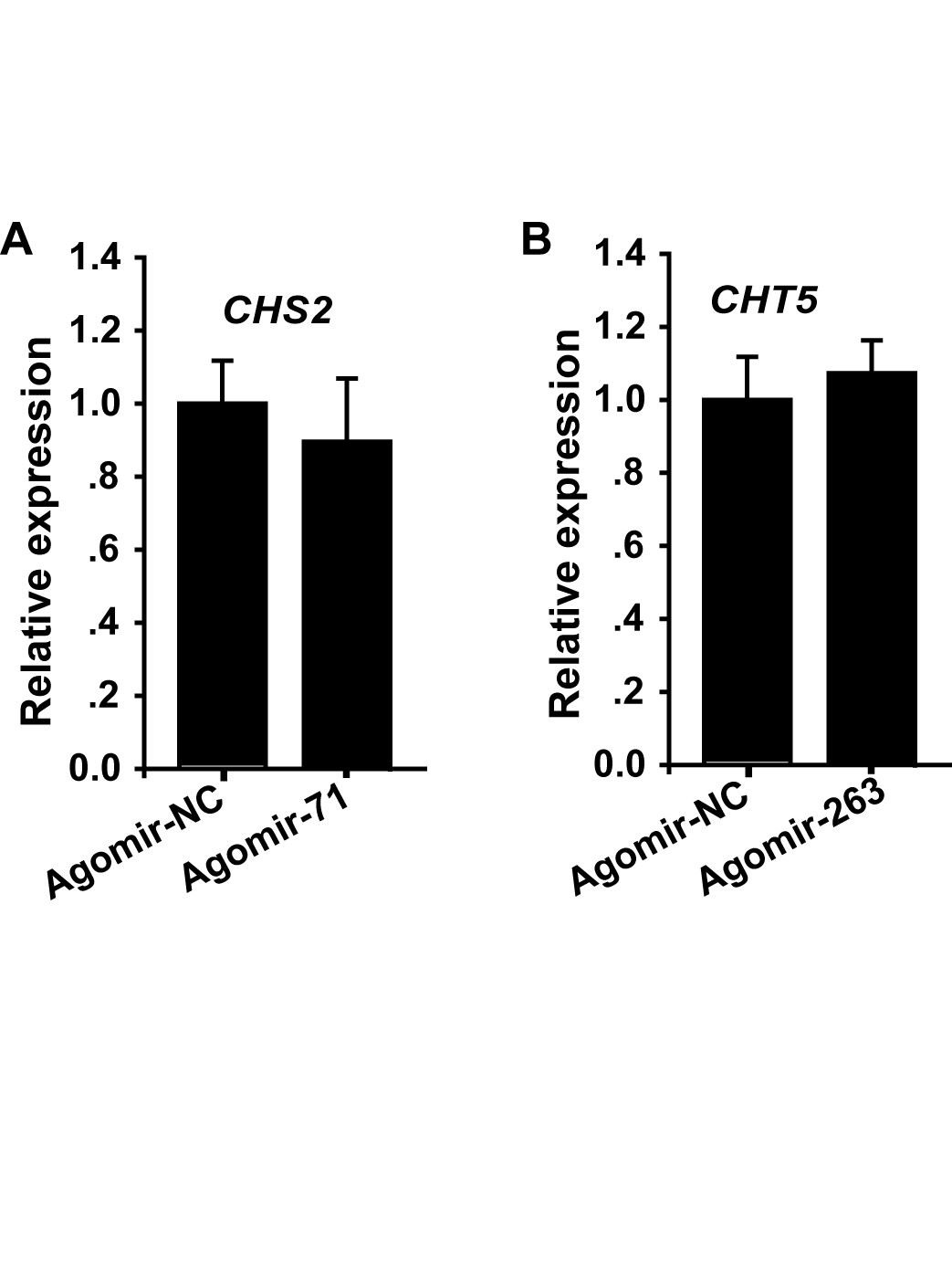

Supplement: S6 Fig — (A) CHS2 expression was quantified using qRT-PCR 48 h after treatment of locusts with 210 pmol agomir-71. (B) CHT5 expression was quantified using qRT-PCR 48 h after treatment of locusts with 210 pmol agomir-263. The data are presented as means ± SEM (n = 6). *p < 0.05; **p < 0.01. (TIF) [file pgen.1006257.s006.tif]

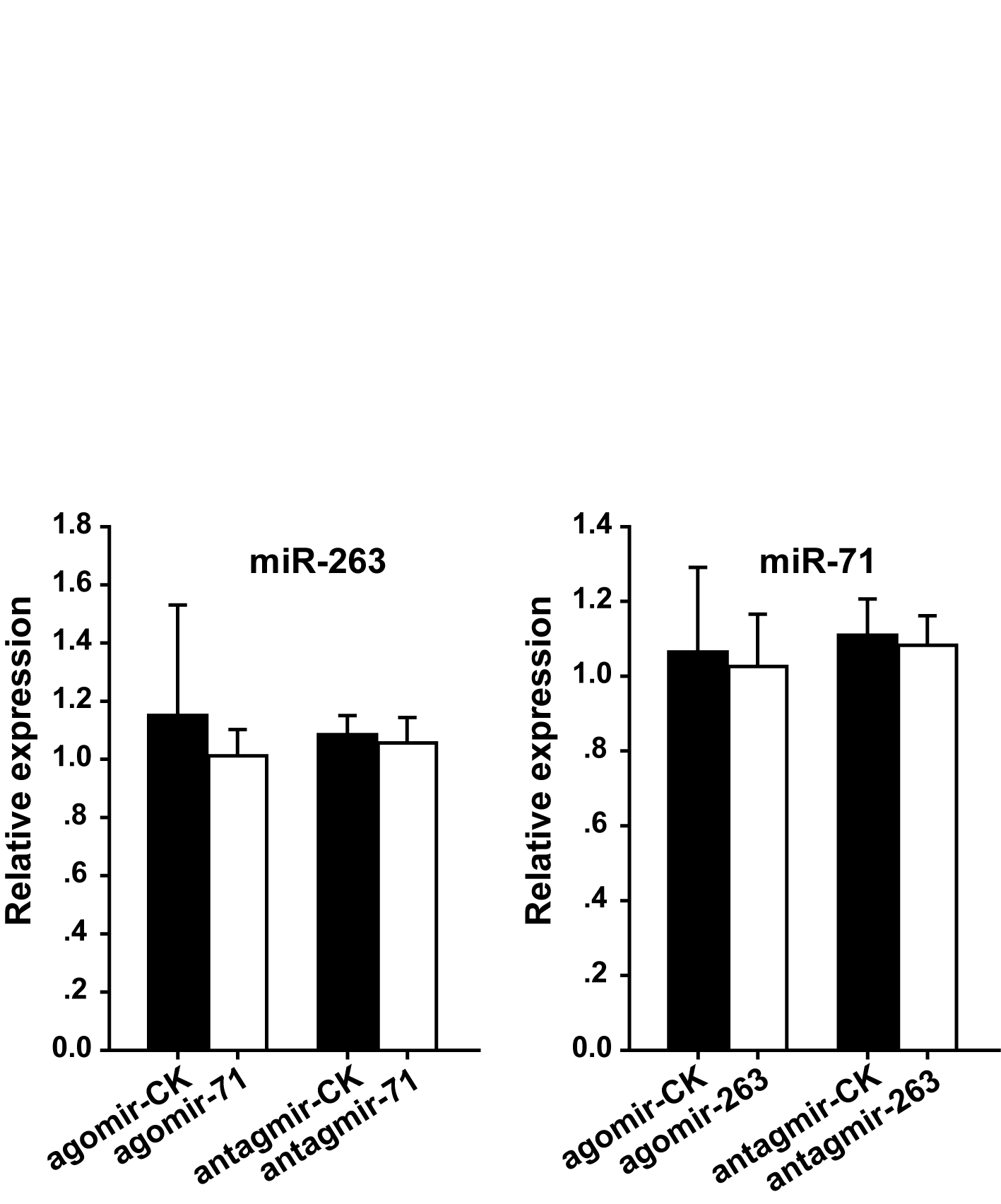

Supplement: S7 Fig — (A) The expression levels of miR-263 were quantified using qPCR 48 h after the locusts were treated with 210 pmol of agomir- and antagomir-71. (B) The expression levels of miR-71 were quantified using qPCR 48 h after the locusts were treated with 210 pmol of agomir- and antagomir-263. All data are presented as means ± SEM (n = 6). (TIF) [file pgen.1006257.s007.tif]

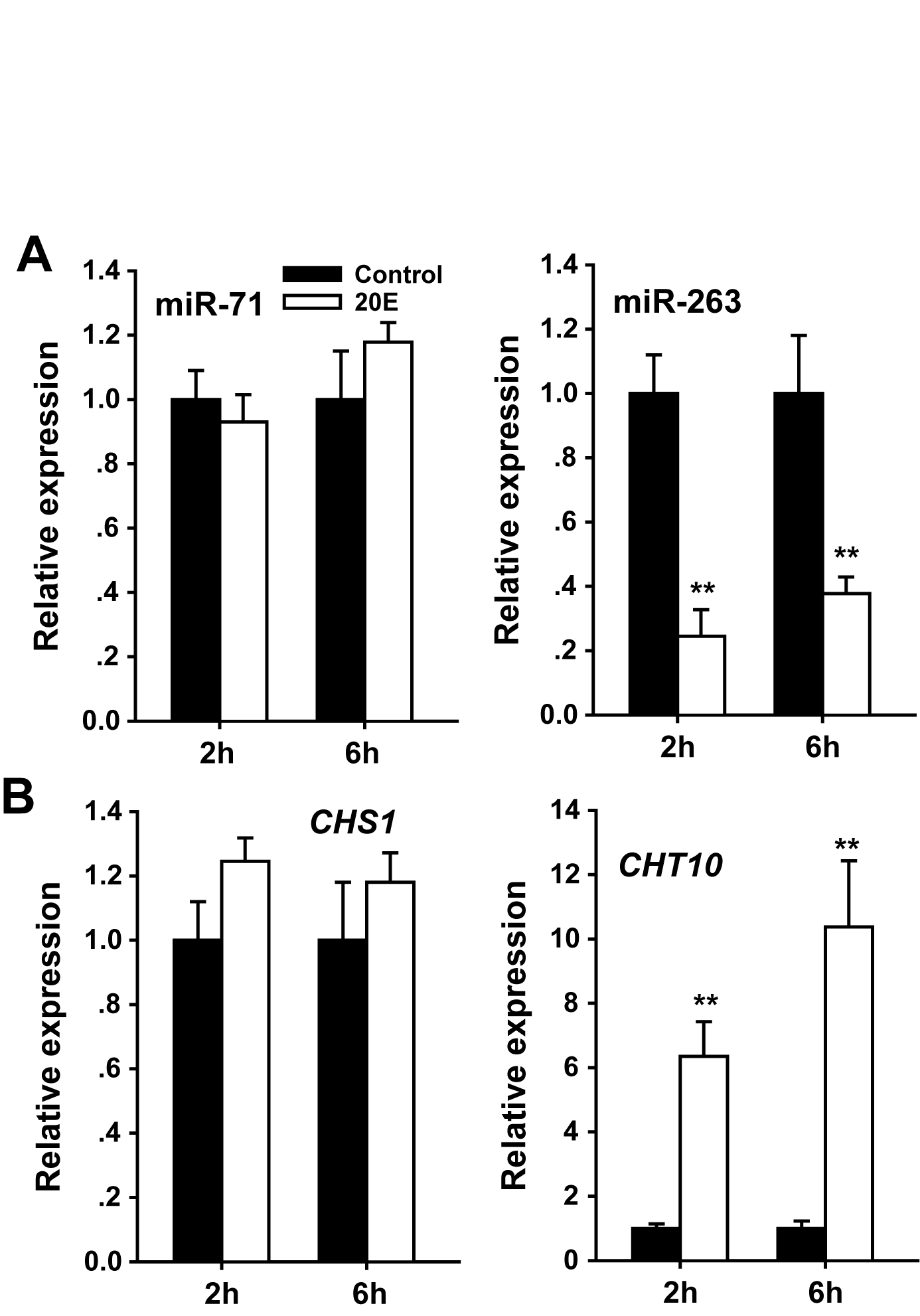

Supplement: S8 Fig — (A) miR-71 and miR-263 expression was quantified using qRT-PCR 2 h and 6 h after the locusts were treated with 20E. (B) The expression levels of CHS1 and CHT10 were quantified using qRT-PCR 2 h and 6 h after the locusts were treated with 20E. The data are presented as means ± SEM (n = 6). **p < 0.01. (TIF) [file pgen.1006257.s008.tif]
